# Medical Prize—Expectant Medicine

**Published:** 1865-11

**Authors:** 


Medical Prize—Expectant Medicine.—One hundred dollars have been placed in the Treasury of the Massachusetts Medical Society, to be offered by the Councillors as a prize for the best dissertation on the following subject, the award to be made by a committee consisting of the President of the Society and four Fellows named by him:

"Expectant Medicine—the extent to which it is practised at the present day, and the modes in which it is disguised or counterfeited."

Essays must be forwarded to the Chairman of the Committee on or before October 1st, 1866, each with a sealed envelope containing the name of its author, in the usual way.

AUGUSTUS A. GOULD,
*Chairman of Committee.*

*Boston, October*, 1865.

In accordance with the above announcement, the following committee has been appointed, namely: Dr. Henry J. Bigelow, Dr. Samuel L. Abbot, Dr. Calvin Ellis and Dr. David W. Cheever.

It will be seen that the terms of the subject cover all cases of honest delusion or wilful fraud in the treatment of disease, in all the forms of excessive medication or the infinitesimal dilutions of homœopathy. As the prize is open to all competitors, we hope that our distant professional brethren may be induced to compete for it.—*Boston Med. & Surg. Journal.*

